# Contact aspiration and stent retriever versus stent retriever alone following mechanical thrombectomy for patients of acute ischemic stroke: A recanalization success analysis

**DOI:** 10.1016/j.clinsp.2023.100262

**Published:** 2023-08-24

**Authors:** Xiaowei Xu, Xiangzhong Shao, Jian Cao, Xiaoyong Huang, Lin Li

**Affiliations:** Department of Neurology, Haian People's Hospital, Haian, Jiangsu, China

**Keywords:** Acute ischemic stroke, Contact aspiration, Reperfusion, Stent retriever catheters, Thrombectomy, Thrombolysis in cerebral infarction

## Abstract

**Objective:**

Second-generation catheters used in mechanical thrombectomy have different advantages and disadvantages. The objective of this study was to evaluate the effectiveness and safety of the combination of contact aspiration and stent retriever technique on the rate of reperfusion after mechanical thrombectomy for large vessel occlusion.

**Methods:**

Patients who underwent contact aspiration alone (CAA cohort, n = 150), stent retriever alone (SRA cohort, n = 129), or combined contact aspiration and stent retriever (CSR cohort, n = 122) techniques following mechanical thrombectomy were included in the analysis. A balloon guide catheter was used for all thrombectomies. Digital subtraction angiography was used to identify thrombolysis in cerebral infarction.

**Results:**

The number of patients with thrombolysis in cerebral infarction score of ≥ 2c (near complete or complete antegrade reperfusion) was significantly higher in the CSR cohort than those in the CAA cohort (101 [83%] vs. 90 [60%], p < 0.0001) and those of SRA cohort (101 [83%] vs. 77 [59%], p = 0.0001). Arterial perforation was higher in patients in the CSR cohort than in those in the CAA (p < 0.0001) and SRA (p = 0.015) cohorts. Intracerebral hemorrhage was lower in patients in the CSR cohort than in those in the CAA (p = 0.0001) and SRA (p = 0.0353) cohorts. All-cause mortality at 1 year was fewer in the CSR cohort than in the CAA cohort (p = 0.018).

**Conclusions:**

The combination of thrombo aspiration by large bore aspiration catheter and stent retriever is the most effective technique but has some related risks.

**Level of evidence:**

IV.

**Technical efficacy stage:**

1.

## Highlights

• A stent-assisted thrombectomy can use in symptomatic intracranial hemorrhage.
• Contact aspiration plus stent retriever catheters have high chances of reperfusion.
• Contact aspiration + stent retriever has fewer disease-related adverse effects.
• The reperfusion parameter should be used to evaluate mechanical thrombectomy.
• Failure attempts increased procedural time and procedural-adverse effects.

## Introduction

Mechanical thrombectomy is generally preferred for better outcomes than medical therapy in conditions of acute ischemic stroke due to large-vessel occlusion(s).^1^ Second-generation catheters, such as contact aspiration catheters or stent retrievers, are widely used in mechanical thrombectomy because large-vessel occlusion(s) are generally present in the anterior circulation.^2^ The American Heart Association Stroke Council,^3^ European Stroke Organization,^4^ and China 2018–2019^5^ acute ischemic stroke guidelines allow all types of mechanical thrombectomy devices (contact aspiration catheters, stent retrievers, or both). However (thrombectomy procedure) should have the highest reperfusion rate because the reperfusion score is always reported less than 60%.^1^,^2^,^6^

A stent retriever technique may have higher rates of distal emboli and fewer rates of symptomatic hemorrhage in patients after thrombectomy than the contact aspiration technique, but a contact aspiration technique may have fewer chances of embolization in new territories and symptomatic intracranial hemorrhage.^7^ A combination of contact aspiration and a stent retriever, a second-generation catheter, reported a high rate of reperfusion after mechanical thrombectomy due to a synergistic combination of the two techniques.^8^, ^9^, ^10^, ^11^ However, in the initial stage of mechanical thrombectomy, the effects of a synergistic combination of the two techniques have not yet been well established.

The objective of this observational study was to evaluate the effectiveness and safety of the combination of contact aspiration and stent retriever on the rate of reperfusion after mechanical thrombectomy for large-vessel occlusion(s).

## Materials and methods

### Ethics approval and consent to participate

The designed protocol (Approval nº HPH15421 dated August 15, 2021) was approved by the Haian People's Hospital Review Board and Chinese Stroke Society. This study followed the laws of China and the V2008 Declaration of Helsinki. This was a retrospective study. Therefore, registration in the Chinese trial registry and patient consent were waived by the Haian People's Hospital Review Board and the Chinese Stroke Society.

### Inclusion criteria

Patients who underwent mechanical thrombectomy for large-vessel occlusion(s) were included in the analysis.

### Exclusion criteria

Patients with serious or fatal comorbidities (e.g., disabilities) that could affect postoperative outcomes were excluded from the study. Patients without a balloon-guiding catheter were excluded from the analysis.

### Mechanical thrombectomy

Under general anesthesia, an arterial puncture was performed to initiate the procedure. A balloon-guide catheter was used. The patient underwent contact aspiration after a mechanical thrombectomy. However, after three failed attempts at contact aspiration, the patients underwent the stent retriever technique. In patients with three failed attempts of contact aspiration and three failed attempts of stent retriever, a combination of contact aspiration and stent retriever following mechanical thrombectomy was performed. Two-thirds of the stent retriever was deployed to the clot and one-third was deployed within the clot. The consulting surgeon(s) decided to use a second-generation catheter (contact aspiration /or stent retriever). A tissue plasminogen activator was not administered in any patient. The selection of a second-generation catheter(s) for the mechanical thrombectomy technique depended on the location of the thrombosis, as well as numerous other anatomic considerations. The ADAPT technique (a direct aspiration first-pass technique) was preferred for attaching the aspiration catheter to a syringe in patients who underwent contact aspiration after mechanical thrombectomy.

### Sample size calculations

The sample size was calculated based on the assumption that included patients might have a minimum of 25% of catheterization failures after mechanical thrombectomy. Additionally, at 80% power calculation and 95% of Confidence Interval (95% CI), the sample size (minimum patients required in each cohort) was 100.

### Outcome measures

Data regarding demographic and clinical characteristics, stroke characteristics, neurological and functional examinations, quality of life score, thrombolysis in cerebral infarction, and safety outcomes were collected from patient records and analyzed.

### National Institutes of Health Stroke Scale (NIHSS) score

It represents a neurological deficiency in 11 categories. The total score is 42. A score of 0 indicates no symptoms and 42 indicates severe neurological deficiency or death.^1^

### Neurological and functional examinations

Regions of hemorrhagic transformation (intracranial hemorrhage) were evaluated according to the European Cooperative Acute Stroke Study III classification.^12^ Any neurological deterioration was assessed using non-contrast computed tomography. Neurological and functional outcomes were evaluated 1d after mechanical thrombectomy.

### Modified Rankin scale

The modified Rankin scale score was administered by a trained nurse (minimum 3 years of experience) to evaluate the quality-of-life score. The quality-of-life score was evaluated pre-thrombectomy and 6 months after thrombectomy. 0, no neurological symptoms; 5, severe disability; 6, death.^13^

### Alberta Stroke Program Early Computed Tomography Score (ASPECTS)

The extent of the stroke was measured using the ASPECTS. Scores ranged from 0 to 10. Higher scores indicate lower early ischemic changes.^1^

### Thrombolysis in cerebral infarction

Digital subtraction angiography was used to identify thrombolysis in cerebral infarction. The score was rated as 0, no perfusion; 1, antegrade perfusion with limited distal branch filling; 2a, antegrade perfusion with less than half distal branch filling; 2b, antegrade perfusion with more than half distal branch filling; 2c, near complete antegrade perfusion; and 3, complete antegrade perfusion.^14^

### Safety outcomes

Magnetic resonance imaging and/or non-contrast computed tomographic images of the brain were used to evaluate disease-related adverse effects such as symptomatic and asymptomatic intracerebral hemorrhage (these were repeated during the follow-up period). The mortality rate was also evaluated. Procedure-related adverse effects, such as arterial perforation, embolization in a new territory, arterial dissection, subarachnoid hemorrhage, and spasms, were evaluated. Safety outcomes were evaluated for 1-year after thrombectomy. The time schedule of the visit during the follow-up period was at every month.

### Statistical analysis

InStat 3.01 GraphPad Software (San Diego, CA, USA) was used for the statistical analysis. Categorical data were analyzed using Fisher's exact test or Chi-Square test (χ^2^-test). χ^2^ test followed by Bonferroni correction was performed when required. No linear continuous or ordinal data were analyzed using the Kruskal-Wallis” Wallis’ test. Bartlett's test was used to check whether the variables among the groups were linear. Gaussian distributions were tested using Kolmogorov and Smirnov methods. Dunn's multiple comparison test was used for *post hoc* analysis. All results were considered significant if the p-value was less than 0.05.

## Results

### Study population

**Fig. 1:**
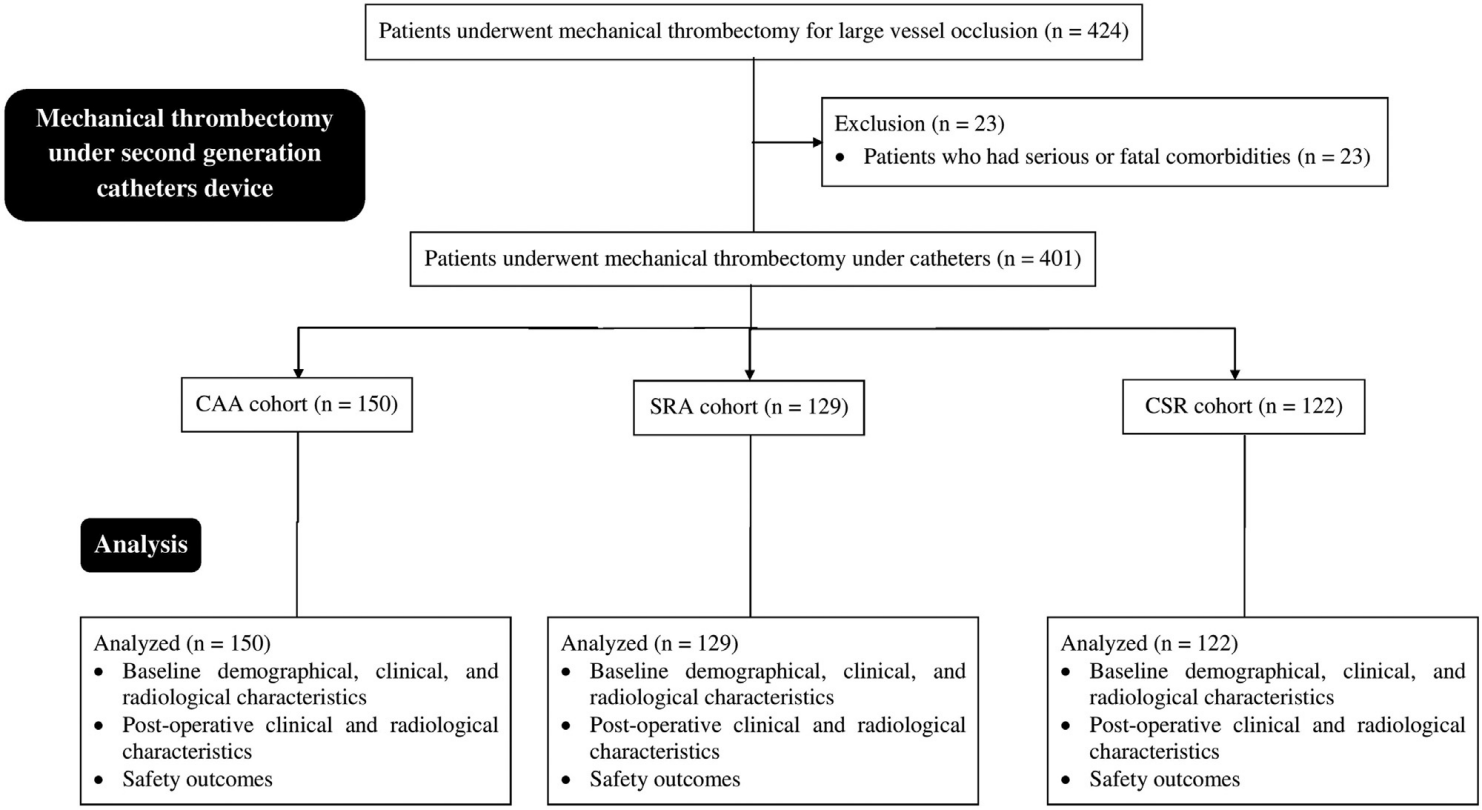
The summary chart of the study. CAA cohort, Patients were subjected to contact aspiration alone catheters-assisted mechanical thrombectomy; SRA cohort, Patients were subjected to stent retriever alone catheters-assisted mechanical thrombectomy after three failed attempts of contact aspiration catheters-assisted mechanical thrombectomy; CSR cohort, Patients were subjected to combined contact aspiration and stent retriever catheters-assisted mechanical thrombectomy after three failed attempts of contact aspiration catheters-assisted mechanical thrombectomy and stent retriever catheters-assisted mechanical thrombectomy each.

From January 15, 2017, to March 17, 2019, a total of 424 patients were admitted to the Department of Emergency of the Haian People's Hospital, Haian, Jiangsu, China for mechanical thrombectomy due to large vessel occlusion(s). A total of 23 patients had fatal comorbidities that could affect postoperative outcomes. Therefore, data from these patients were excluded. Among 401 patients who underwent mechanical thrombectomy due to large vessel occlusion(s), 150 were subjected to contact aspiration alone following mechanical thrombectomy (CAA cohort). A total of 129 patients were subjected to stent retriever therapy following mechanical thrombectomy (SRA cohort). A total of 122 patients were subjected to combined contact aspiration and stent retriever therapy following mechanical thrombectomy (CSR cohort). A summary of this study is shown in Fig. 1.

### Demographical and clinical characteristics

Demographic and clinical characteristics and medical history of the patients at the time of hospital admission were comparable (p > 0.05, Kruskal-Wallis’ test or χ^2^-test, Table 1).

**Table 1:** Demographical and clinical characteristics and medical history of patients before thrombectomy at the time of hospital admission.

**Parameters**		**Total**	**Cohorts**			**Comparisons among cohorts**
			**CAA**	**SRA**	**CSR**	
**Second generation catheter**		**Contact aspiration or/ and Stent retriever**	**Contact aspiration alone**	**Stent retriever alone**	**Contact aspiration + Stent retriever**	
Numbers of patients		401	150	129	122	p-value
Age (years)		64 (68–58)	65 (71–57)	64 (66–59)	62 (67–56)	0.0873 (Kruskal-Wallis’ test)
Gender	Male	220 (55)	80 (53)	70 (54)	70 (57)	0.7898 (χ^2^-test)
	Female	181 (45)	70 (47)	59 (46)	52 (43)	
Hypertensive patients		199 (50)	67 (45)	62 (48)	70 (57)	0.1036 (χ^2^-test)
Diabetes		89 (22)	33 (22)	29 (22)	27 (22)	0.9952 (χ^2^-test)
Hypercholesterolemia		138 (34)	51 (34)	44 (34)	43 (35)	0.9733 (χ^2^-test)
Smoking	Current	43 (11)	15 (10)	14 (11)	14 (12)	0.9737 (χ^2^-test)
	Previous	50 (12)	17 (11)	17 (13)	16 (13)	
	Non-smoker	308 (77)	118 (79)	98 (76)	92 (75)	
Coronary artery disease		34 (8)	11 (7)	11 (9)	12 (10)	0.762 (χ^2^-test)
History of transient ischemic attack or stroke		73 (18)	30 (20)	16 (12)	27 (22)	0.1052 (χ^2^-test)
History of antiplatelet medication		168 (42)	61 (41)	58 (45)	49 (40)	0.6903 (χ^2^-test)
History of anticoagulant medication		92 (23)	32 (21)	30 (23)	30 (25)	0.8129 (χ^2^-test)

Variable depicted as median (Q3–Q1) or frequency (percentage).

A p < 0.05 was considered significant.

### Stroke and thrombectomy characteristics

#### Stroke characteristics

At the time of hospital admission, systolic blood pressure, NIHSS score, modified Rankin scale score, and ASPECTS of patients among the cohorts were comparable (p > 0.05, Kruskal-Wallis’ test, Table 2).

**Table 2:** Pre-thrombectomy stroke characteristics of patients.

**Parameters**	**Total**	**Cohorts**			**Comparisons among cohorts**
		**CAA**	**SRA**	**CSR**	
**Second generation catheter**	**Contact aspiration or/ and Stent retriever**	**Contact aspiration alone**	**Stent retriever alone**	**Contact aspiration + Stent retriever**	
Numbers of patients	401	150	129	122	p-value
SBP (mmHg)	156 (163–146)	157 (162–149)	158 (167–146)	153 (163–142)	0.057
Pre-thrombectomy NIHSS score	18 (21–16)	19 (21–16)	18 (20–17)	18 (20–16)	0.0906
Pre-thrombectomy the modified Rankin scale score	3 (4–2)	3 (4–2)	3 (4–2)	3 (4–2)	0.7863
ASPECTS	7 (8–6)	7 (8–6)	7 (8–6)	7	0.0791

Variable depicted as median (Q3–Q1).

SBP, Systolic Blood Pressure; NIHSS, National Institutes of Health Stroke Scale (0 dedicates no symptoms and 42 dedicates severe neurological deficiency or death); ASPECTS, Alberta Stroke Program Early Computed Tomography Score.

The modified Rankin scale score: 0, no neurological symptoms; 5, severe disability.

Kruskal-Wallis’ test was used for statistical analysis purposes.

A p < 0.05 was considered significant.

#### Occlusion sites characteristics

When occlusion was present in the middle cerebral artery M1, contact aspiration alone failed in most cases, and a stent retriever alone or in combination with a stent retriever catheter was preferred. When occlusion was present in the middle cerebral artery M2, contact aspiration alone was used, except in cases of other complications (Table 3).

**Table 3:** Occlusion site characteristics of patients.

**Parameters**	**Total**	**Cohorts**			**Comparisons among cohorts**
		**CAA**	**SRA**	**CSR**	
**Second generation catheter**	**Contact aspiration or/ and Stent retriever**	**Contact aspiration alone**	**Stent retriever alone**	**Contact aspiration + Stent retriever**	
Numbers of patients	401	150	129	122	p-value
Middle cerebral artery M1	260 (66)	90 (60)	85 (66)	85 (70)	0.2041
Middle cerebral artery M2	40 (10)	20 (13)	10 (8)	10 (8)	0.2205
Intracranial internal carotid artery	53 (13)	22 (15)	19 (15)	12 (10)	0.4174
Tandem extracranial	22 (5)	9 (6)	6 (4)	7 (6)	0.876
Tandem intracranial	9 (2)	3 (2)	3(2)	3 (2)	0.9654
Extracranial internal carotid artery	9 (2)	3 (2)	4 (3)	2 (2)	0.7133
Anterior cerebral artery	7 (2)	3 (2)	2 (2)	3 (2)	0.876

Variable depicted as frequency (percentage).

χ^2^-test was used for statistical analysis purposes.

A p < 0.05 was considered significant.

#### Procedural characteristics

A balloon-guide catheter was the choice for all thrombectomies. The time from arterial puncture to clot contact was the same in all cohorts. However, procedural time was higher in the SRA cohort than in the CAA cohort (p<0.001, Kruskal-Wallis test/Dunn's multiple comparisons test). In addition, procedural time was higher in the CSR cohort than in the CAA cohort (p<0.001, Kruskal-Wallis ‘test/Dunn's multiple comparisons test) and those for the SRA cohort (p<0.001, Kruskal-Wallis’ test/Dunn's multiple comparisons test). Details of the stroke and thrombectomy procedural characteristics are presented in Table 4.

**Table 4:** Thrombectomy procedural characteristics of patients.

**Parameters**	**Total**	**Cohorts**			**Comparisons among cohorts**
		**CAA**	**SRA**	**CSR**	
**Second generation catheter**	**Contact aspiration or/and Stent retriever**	**Contact aspiration alone**	**Stent retriever alone**	**Contact aspiration + Stent retriever**	
Numbers of patients	401	150	129	122	p-value
Balloon guide catheter use	359 (90)	132 (88)	112 (86)	115 (94)	0.1166 (χ^2^-test)
Time from an arterial puncture to clot contact (min)	31 (35–26)	31 (35–26)	31 (34–26.5)	30 (34–25)	0.3218 (Kruskal-Wallis’ test)
Intracranial hemorrhage	33 (8)	12 (8)	11 (9)	10 (8)	0.9872 (χ^2^-test)
Procedural time (min)	55 (64–49.5)	50 (54–48)	56 (63–52)	71 (82–57)	<0.0001 (Kruskal-Wallis’ test)

Variable depicted as median (Q3–Q1) or frequency (percentage).

A p < 0.05 was considered significant.

### Thrombolysis in cerebral infarction

The individual thrombolysis scores in cerebral infarction for the CAA and SRA cohorts were not different from those of the CSR cohort (p > 0.05, *χ*^2^-test with Bonferroni's correction, Table 5). However, the number of patients with thrombolysis in cerebral infarction scores of 2c or more (2c or 3; near-complete or complete antegrade reperfusion) was significantly higher in the CSR cohort than in the CAA (p < 0.0001, *χ*^2^-test with Bonferroni's correction) and SRA (p = 0.0001, *χ*^2^-test with Bonferroni's correction) cohorts. In addition, the number of patients with thrombolysis in cerebral infarction score of ≥ 2b (2b, 2c, or 3; antegrade reperfusion with more than half distal branch filling or near complete antegrade reperfusion or complete antegrade reperfusion) was significantly higher in the CSR cohort than in the CAA (p = 0.0026, *χ*^2^-test with Bonferroni's correction) and SRA (p = 0.0011, *χ*^2^-test with Bonferroni's correction) cohorts.

**Table 5:** Thrombolysis in cerebral infarction after mechanical thrombectomy.

**Score**	**Total**	**Cohorts**					**Comparison between CAA and SRA cohorts**
		**CSR**	**CAA**		**SRA**		
**Second generation catheter**	**Contact aspiration or/ and Stent retriever**	**Contact aspiration + Stent retriever**	**Contact aspiration alone**		**Stent retriever alone**		
Numbers of patients	401	122	150	bp-value	129	bp-value	p-value
0	05 (1)	0 (0)	02 (1)	0.571	02 (2)	0.5025	0.8791
1	35 (9)	5 (4)	16 (11)	0.0734	14 (11)	0.0745	0.9601
2a	43 (11)	7 (6)	19 (13)	0.0844	17 (13)	0.0736	0.8989
2b	51 (13)	9 (7)	23 (15)	0.0663	19 (15)	0.073	0.888
2c	179 (44)	65 (53)	61 (41)	0.0509	53 (41)	0.0992	0.9435
3	89 (22)	36 (30)	29 (19)	0.0697	24 (18)	0.0606	0.9987
≥2c	268 (66)	101 (83)	90 (60)^a^	<0.0001	77 (59)^a^	0.0001	0.958
≥2b	319 (81)	110 (90)	113 (75)^a^	0.0026	96 (74)^a^	0.0011	0.9703

Variable depicted as frequency (percentage).

a Significantly fewer than that of patients of the CSR cohort.

b Respect to CSR. 0, No reperfusion; 1, Antegrade reperfusion with limited distal branch filling; 2a, Antegrade reperfusion with less than half distal branch filling; 2b, Antegrade reperfusion with more than half distal branch filling; 2c, Near complete antegrade reperfusion, and 3, Complete antegrade reperfusion. The Chi-Square test with Bonferroni's correction was used for statistical analysis. A p < 0.05 was considered significant.

### Outcome measures

The NIHSS score 1d after thrombectomy and the modified Rankin scale score 6 months after thrombectomy of patients were comparable among cohorts (p > 0.05, Kruskal-Wallis’ test, Table 6).

**Table 6:** Outcome measures of patients after mechanical thrombectomy.

**Score**	**Total**	**Cohorts**			**Comparisons among cohorts**
		**CSR**	**CAA**	**SRA**	
**Second generation catheter**	**Contact aspiration or/and Stent retriever**	**Contact aspiration + Stent retriever**	**Contact aspiration alone**	**Stent retriever alone**	
Numbers of patients	401	122	150	129	p-value
NIHSS score after 1-day of thrombectomy	18 (21–16)	19 (21–16)	18 (20–17)	18 (20–16)	0.1255
The modified Rankin scale score after 6-months of thrombectomy	3 (4–2)	3 (4–2)	3 (4–2)	3 (4–2)	0.7494

Variable depicted as median (Q3–Q1).

NIHSS, National Institutes of Health Stroke Scale (0 dedicates no symptoms and 42 dedicates severe neurological deficiency or death).

The modified Rankin scale score: 0, No neurological symptoms; 5, Severe disability, 6, Post-operative death in surgical intensive care unit/death during surgeries.

Kruskal-Wallis’ test was used for statistical analysis purposes.

A p < 0.05 was considered significant.

### Safety outcomes

#### Procedural-related worse effects

Total procedure-related adverse effects were higher in patients who had contact aspiration plus stent retriever catheter than in those who had contact aspiration alone (p < 0.0001, χ2-test followed by Bonferroni corrections) and stent retrievers alone (p = 0.0005, χ2-test followed by Bonferroni corrections). Arterial perforation was higher in patients who had contact aspiration plus stent retriever catheter than in those who had contact aspiration alone (p = 0.0002, χ2-test followed by Bonferroni corrections) and stent retrievers alone (p = 0.0181, χ2-test followed by Bonferroni corrections). Embolization in a new territory was also higher in patients who had contact aspiration plus a stent retriever catheter than in those who had contact aspiration alone (p = 0.0383, χ2-test followed by Bonferroni corrections). Embolization in a new territory was also higher in patients who had contact aspiration plus a stent retriever catheter than in those who had a stent retriever alone, but the difference was not statistically significant (p = 0.332, χ2-test followed by Bonferroni corrections). Total procedure-related adverse effects were higher in patients who had stent retriever alone catheter than those who had contact aspiration alone (p = 0.0065, χ2-test followed by Bonferroni corrections). The other procedural worse effects were comparable among patients (p>0.05, Fisher's exact test, Table 7).

**Table 7:** Procedural-related adverse effects of patients during 1-years after the thrombectomy.

**Adverse effect**	**Total**	**Cohorts**					**Comparison between CAA and SRA cohorts**
		**CSR**	**CAA**		**SRA**		
**Second generation catheter**	**Contact aspiration or/and Stent retriever**	**Contact aspiration + Stent retriever**	**Contact aspiration alone**		**Stent retriever alone**		
Numbers of patients	401	122	150	bp-value	129	bp-value	p-value
Arterial perforation	32 (8)	20 (16)	4 (3)^a^	0.0002	8 (6)^a^	0.0181	0.2481
Embolization in a new territory	32 (8)	15 (12)	7 (5)^a^	0.0383	10 (8)	0.332	0.4104
Arterial dissection	21 (5)	10 (8)	4 (3)	0.0756	7 (5)	0.5431	0.383
Subarachnoid hemorrhage	18 (5)	9 (7)	3 (2)	0.0642	6 (5)	0.5194	0.3629
Spasms	15 (4)	7 (6)	3 (2)	0.1918	5 (4)	0.6928	0.5644
Total	118 (30)	61 (50)	21 (14)^a^^,^^c^	<0.0001	36 (28)^a^	0.0005	0.0065

Variable depicted as frequency (percentage).

Patients have one or more procedure-related adverse effects.

The *χ*^2^-test followed by Bonferroni corrections was used for statistical analysis.

a Significantly fewer than that of patients of the CSR cohort.

b Respect to CSR. A p < 0.05 was considered significant.

c Significantly fewer than that of patients of the SRA cohort.

#### Disease-related adverse effects

Disease-related adverse effects were fewer in patients who had contact aspiration plus stent retriever catheter than in those who had contact aspiration alone (p < 0.0001, for all, χ^2^-test with Bonferroni's correction) and stent retriever alone (p < 0.0001, for all, χ^2^-test with Bonferroni's correction). Symptomatic intracerebral hemorrhage was lower in patients who had contact aspiration plus a stent retriever catheter than in those who had contact aspiration alone (p = 0.0011, χ^2^-test with Bonferroni's correction). Total disease-related adverse effects were fewer in patients who had a stent retriever alone catheter than in those who had contact aspiration alone (p < 0.0001, for all, χ^2^-test with Bonferroni's correction). Symptomatic intracerebral hemorrhage was lower in patients who had a stent retriever catheter alone than in those who had contact aspiration alone; however, the difference was not statistically significant (p = 0.9585, χ^2^-test with Bonferroni's correction). Intracerebral hemorrhage was lower in patients who had contact aspiration plus stent retriever catheter than in those who had contact aspiration alone (p = 0.0001, χ^2^-test with Bonferroni's correction) and stent retriever alone (p = 0.0353, χ^2^-test with Bonferroni's correction). All-cause mortality was lower in patients who had contact aspiration plus stent retriever catheter than in those who had contact aspiration alone (p = 0.018, χ^2^-test with Bonferroni's correction) and stent retriever alone, but the difference was not statistically significant (p = 0.0542, χ^2^-test with Bonferroni's correction). The other disease-related adverse effects of patients were not significant among all cohorts (p > 0.05 for all, χ^2^-test with Bonferroni's correction; data are not presented in the report). The disease-related adverse effects are reported in Table 8.

**Table 8:** Disease-related adverse effects of patients during 1-years after the thrombectomy.

**Adverse effect**	**Total**	**Cohorts**					**Comparison between CAA and SRA cohorts**
		**CSR**	**CAA**		**SRA**		
**Second generation catheter**	**Contact aspiration or/ and Stent retriever**	**Contact aspiration + Stent retriever**	**Contact aspiration alone**		**Stent retriever alone**		
Numbers of patients	401	122	150	ap-value	129	ap-value	p-value
Symptomatic intracerebral hemorrhage	112 (28)	22 (18)	55 (37)^b^	0.0011	35 (27)	0.1166	0.1164
Asymptomatic intracerebral hemorrhage	46 (11)	10 (8)	20 (13)	0.25	16 (12)	0.63	0.9585
Intracerebral hemorrhages	158 (39)	32 (26)	75 (50)^b^	0.0001	51 (40)^b^	0.0353	0.103
Total	316 (78)	64 (52)	150 (100)^b^^,^^c^	<0.0001	102 (79)^b^	<0.0001	<0.0001
All-cause mortality within 1 year	153 (38)	35 (29)	65 (43)^b^	0.018	53 (41)	0.0542	0.7968

Variable depicted as frequency (percentage).

The Chi-square test with Bonferroni's correction was used for statistical analysis.

Patients have one or more disease-related adverse effects.

a Respect to CSR.

b Significantly higher than that of the CSR cohort.

c Significantly higher than that of the SRA cohort. A p < 0.05 was considered significant.

## Discussion

The study found that the number of patients with near complete or complete antegrade reperfusion (thrombolysis in cerebral infarction score of ≥ 2c) and the numbers of patients with ≥ 50% reperfusion (thrombolysis in cerebral infarction score of ≥ 2b) were higher in the CSR cohort than those in the CAA and SRA cohorts. The results of reperfusion in the current study were consistent with those of a randomized trial^6^ but were not parallel with those of the ASTER trial^7^ and the ASTER2 trial.^1^ The large sample sizes of the ASTER ^7^ and ASTER2^1^ trials are responsible for type II errors (false negative; contradictory results). In addition, the ASTER ^7^ and ASTER2 ^1^ trials are under power. The patients included in the study have undergone one of these three techniques initially, however patients in the ASTER trial^7^ and the ASTER2 trial ^1^ one of these three techniques was as a rescue measure. Therefore, this technique was applied under different circumstances and difficulty levels in the current study. Patients with acute ischemic stroke who undergo thrombectomy with a balloon guide catheter following contact aspiration plus stent retriever catheters have a high chance of reperfusion after mechanical thrombectomy.

In the current retrospective study, near complete or complete reperfusion (thrombolysis in cerebral infarction score of ≥ 2c) was used as the main outcome measure. Reperfusion is a feasible and early biomarker of treatment success.^6^,^15^,^16^ Reperfusion parameters should be used to evaluate mechanical thrombectomy.

Procedural time and procedure-related adverse effects were higher for mechanical thrombectomy following contact aspiration plus stent retriever catheter than for those who had contact aspiration or stent retriever alone. The results of procedural time and procedure-related adverse effects in the current study were not consistent with those of the ASTER trial ^7^ and the ASTER2 trial.^1^ The reasons for the contradictory results are that the ASTER trial ^7^ and ASTER2 trial ^1^ are randomized studies where the use of a catheter was decided, but in the current study, the choice of the catheter was based on the merits of the situation. Failure of thrombectomy increased procedural time and procedure-related adverse effects.

Disease-related adverse effects, including all-cause mortality, were fewer in patients who underwent mechanical thrombectomy following contact aspiration plus stent retriever catheter than in those who had contact aspiration alone or stent retriever alone. The results of the disease-related adverse effects in the current study were not consistent with those of the ASTER trial ^7^ and the ASTER2 trial.^1^ The large sample sizes of the ASTER ^7^ and ASTER2 ^1^ trials are responsible for type II errors (contradictory results; false negatives). Second, the ASTER^7^ and ASTER2^1^ trials are studies under power. Third, in the current study, adverse effects were reported in a site-specific manner, while in the ASTER ^7^ and ASTER2 ^1^ trials, adverse effects were evaluated blindly. Fourth, the follow-up time in the ASTER ^7^ and ASTER2 ^1^ trials was 90 days, and the follow-up time in the current study was 1 year after mechanical thrombectomy. Mechanical thrombectomy following contact aspiration using a stent retriever catheter has fewer disease-related adverse effects.

Approximately 50% of patients had hemorrhagic complications (including subarachnoid, symptomatic intracerebral, and asymptomatic intracerebral hemorrhage). Thrombolysis, a firm cofounder of hemorrhagic complications, is used before thrombectomy.

In the limitations of the study, for example, experience and expertise have a great impact on the success rate of mechanical thrombectomy.^1^,^17^ However, the experience and expertise of the operators were not reported or discussed in the current study. The other limitations are the retrospective nature of the analysis and the lack of dynamic studies. Although disease-related adverse effects and better reperfusion were reported in patients who underwent mechanical thrombectomy following contact aspiration plus stent retriever catheter, the study failed to evaluate favorable outcomes such as 1d post-procedure NIHSS score, 6-months post procedure the modified Rankin scale score, and an individual thrombolysis score in patients with cerebral infarction compared to patients who underwent mechanical thrombectomy following contact aspiration alone or mechanical thrombectomy following stent retriever catheter alone. The study also failed to justify the same maximum blood pressure in all cohorts and the same range of time from an arterial puncture to clot contact (min) in all cohorts. A total of 23 patients with serious or fatal comorbidities were excluded from the analyses. However, no indication of pre-stroke disability or comorbidity was observed. Neither for this cohort nor for those that were excluded. Information on stroke etiology in this cohort was lacking. There is a lack of information on the precise location of the embolus in the ICA or M1 (prox., dist.) or M2, MCA, which is well known to significantly determine the thrombectomy procedure.

## Conclusions

This is a study of the treatment success of contact aspiration and stent retriever *versus* stent retriever alone. Balloon-guided contact aspiration plus stent retriever catheters following thrombectomy have a high chance of reperfusion for large-vessel occlusion. In addition, contact aspiration plus stent retriever catheter-assisted mechanical thrombectomy has fewer disease-related adverse effects and mortality at 1-year follow-up after mechanical thrombectomy. Contact aspiration with stent retriever catheters following mechanical thrombectomy may pose a risk of procedure-related adverse effects. In the current single-center retrospective case series, there are likely many cases in which one or the other of the techniques failed, and the two techniques were used in combination. Therefore, parsing the results and comparing them is of little utility in guiding the performance of future mechanical thrombectomies.

## Authors’ contributions

All the authors have read and approved the manuscript for publication. XX was the project administrator and contributed to the supervision, methodology, resources, and literature review. XS contributed to the investigation, validation, methodology, literature review, and the software used in the study. JC contributed to the conceptualization, methodology, visualization, literature review, and supervision of the study. XH contributed to formal analysis, data curation, methodology, software, and literature review. LL contributed to the literature review, resources, and methodology of the study, and drafted and edited the manuscript for intellectual content. All authors agree to be responsible for all aspects of the work, ensuring its integrity and accuracy.

## Funding

This research did not receive any specific grants from funding agencies in the public, commercial, or not-for-profit sectors.

## Availability of data and materials

The datasets generated and/or analyzed during the current study are available from the corresponding author upon reasonable request.

## Declaration of Competing Interest

The authors declare no conflicts of interest or any other competing interests regarding the results and/or discussion reported in the research.
